# The GC/MS Analysis of Volatile Components Extracted by Different Methods from *Exocarpium Citri Grandis*

**DOI:** 10.1155/2013/918406

**Published:** 2013-11-17

**Authors:** Zhisheng Xie, Qundi Liu, Zhikun Liang, Mingqian Zhao, Xiaoxue Yu, Depo Yang, Xinjun Xu

**Affiliations:** ^1^School of Pharmaceutical Sciences, Guangzhou Higher Education Mega Center, Sun Yat-sen University, No. 132 East Waihuan Road, Guangzhou 510006, China; ^2^Guangdong Technology Research Center for Advanced Chinese Medicine, Guangzhou 510006, China

## Abstract

Volatile components from *Exocarpium Citri Grandis* (ECG) were, respectively, extracted by three methods, that is, steam distillation (SD), headspace solid-phase microextraction (HS-SPME), and solvent extraction (SE). A total of 81 compounds were identified by gas chromatography-mass spectrometry including 77 (SD), 56 (HS-SPME), and 48 (SE) compounds, respectively. Despite of the extraction method, terpenes (39.98~57.81%) were the main volatile components of ECG, mainly germacrene-D, limonene, 2,6,8,10,14-hexadecapentaene, 2,6,11,15-tetramethyl-, (E,E,E)-, and *trans*-caryophyllene. Comparison was made among the three methods in terms of extraction profile and property. SD relatively gave an entire profile of volatile in ECG by long-time extraction; SE enabled the analysis of low volatility and high molecular weight compounds but lost some volatiles components; HS-SPME generated satisfactory extraction efficiency and gave similar results to those of SD at analytical level when consuming less sample amount, shorter extraction time, and simpler procedure. Although SD and SE were treated as traditionally preparative extractive techniques for volatiles in both small batches and large scale, HS-SPME coupled with GC/MS could be useful and appropriative for the rapid extraction and qualitative analysis of volatile components from medicinal plants at analytical level.

## 1. Introduction

*Exocarpium Citri Grandis* (ECG, Huajuhong in Chinese), the dried unripe or ripe fruit peel of *Citrus grandis *Osbeck or *Citrus grandis* Osbeck var. tomentosa Hort, is a well-known traditional Chinese medicine [1]. Since it has been proved to possess the effects of clearing heat and expectoration, regulating the flow of vital energy, and stimulating appetite, ECG has been employed in the treatment of coughing, dyspepsia, nausea, itching of throat, and so forth, which makes it widely used in practice for a long time in China [2–4]. ECG from *Citrus grandis* “tomentosa” (Maojuhong in Chinese) is generally acknowledged to be of better quality than *Citrus grandis (L.)* Osbeck (Guangjuhong in Chinese). ECG from Huazhou city, Guangdong province, whose plant origin is *Citrus grandis* “tomentosa,” is usually considered as the genuine medicinal materials [5]. 

Main phytochemical constituents of ECG were reported as volatile oil, flavonoids, coumarins, and polysaccharides [6–8], among which volatile composition has been investigated worldwide and the essential oil from ECG was broadly used as aroma flavor in food products and flavoring agents to mask the unpleasant tastes of drugs in pharmaceutical industries [9]. Alkene is the major volatile compounds of ECG typically including limonene, pinene, and myrcene [10]. The amount of volatile components of ECG plays a significant role in the quality of crude drug. For example, limonene, one of the principal components of ECG, possesses the antibacteria, anti-inflammatory, and expectorant activity [11]. 

Traditionally, the extraction of volatile components from ECG relied largely on steam distillation (SD) and solvent extraction (SE) at both analytical and preparative levels. However, there have been rare studies on the comparison of volatile components from ECG by different extracting methods. The innovative solid-phase microextraction (SPME) was more rapid, sensitive, and solvent-free compared to traditional methods. SPME was first introduced by Professor Pawliszyn and his coworkers in 1990s and has been extensively adopted in air, water, soil, and food analysis [12]. Typically, the analytes are extracted from a gaseous or liquid sample by absorption in direct-immerse solid-phase microextraction (DI-SPME) or adsorption on headspace solid-phase microextraction (HS-SPME) with a thin polymer coating being fixed to the solid surface of a fiber in an injection needle [13]. Since HS-SPME was nonpolluting to fiber compared with DI-SPME, it was eventually chosen to be compared with SD and SE. Gas chromatography-mass spectrometry (GC/MS) enables compound identification by comparing the obtained mass spectra of the analytes with those of authentic standards from the National Institute of Standards and Technology (NIST) and comparing the retention indices (RIs) with those reported by a previous available study. 

The work stated here is aiming to fill in the blank field through the analysis of volatile components from ECG extracted by SD, HS-SPME, and SE methods individually. GC/MS was employed to identify compounds in the extracted samples. This would reveal how many volatile components they were able to extract and consequently help evaluate the extraction efficiency in addition to sample amount and extraction duration.

## 2. Materials and Methods

### 2.1. Materials and Reagents

ECG (Huazhou, Guangdong, China) was purchased from Caizhilin pharmacy and was authenticated by Dr. Xu. It was ground to a certain particle size (60 meshes) for the follow-up pretreatments. Anhydrous sodium sulphate was provided by Guangzhou Chemical Reagent Factory (Guangzhou, China). Anhydrous ethyl alcohol, diethyl ether, and *n-*hexane were analytically pure and purchased from Damao Chemical Reagents Works (Tianjin, China). 

### 2.2. Steam Distillation Procedure

Thirty-six grams of ECGpowder was suspended in 300 mL of water to collect the volatile oil by steam distillation for 5 h according to Appendix XD of Chinese Pharmacopoeia [14]. A little drop of kelly green oil was diluted with 1 mL of  *n*-hexane and dehydrated by adding adequate anhydrous sodium sulphate. The solution was then centrifuged for 5 minutes to obtain the volatile oil sample. 

### 2.3. HS-SPME Procedure

Divinylbenzene/carboxen/polydimethylsiloxane (DVB/CAR/PDMS, 50/30 *μ*m), which was designed for analytes with a broad range of polarities (suitable for C_2_–C_20_ range) [15, 16], was purchased from Supelco (Supelco Park Bellefonte, Pennsylvania, USA). It was attached in a SPME holder (Supelco) and used to achieve absorption of volatile components as full as possible from ECG for qualitative analysis. The fiber was conditioned prior to use by inserting it to the GC injection port at 280°C for 2 h under 1 mL/min of gas flow. ECG powder (0.2 g) and anhydrous sodium sulphate (0.2 g) were mixed in a 15 mL flat bottom headspace vial which was sealed with a gray butyl headspace stopper and a 20 mm unlined crimp cap using a crimper. SPME fiber was pushed out and exposed to the headspace of the vial for the absorption of the volatile components, with the vial heated at sustained 80°C for 40 min. Finally, the fiber was removed from the vial and analytes were desorbed by exposing the fiber in the injection port of a GC/MS at 250°C for 2 min.

### 2.4. Diethyl Ether Extraction

Three grams of ECG powder was extracted using diethyl ether (1 : 10, w/v) for three times (15 minutes each time) with the assistance of ultrasonic. The obtained turbid solution was filtrated and the solvent of filtrate was removed by rotary evaporation under reduced pressure. Then the extractum was diluted with 1 mL of anhydrous ethyl alcohol:  *n*-hexane (1 : 1, v/v) and was filtered through a 0.22 *μ*m membrane filter. 1 *μ*L of subsequent filtrate was injected to GC/MS for analysis.

### 2.5. GC-MS Analysis and Identification for Volatile Components

The analysis for volatiles in ECG was performed by the GC/MS instrument (Thermo Electron Corporation, USA) equipped with a Finnigan Trace DSQ and an electron impact (EI) ion source. The analytes were separated on a DB-5MS capillary column (30 m × 0.25 mm × 0.25 *μ*m; Agilent, USA) coated with phenyl arylene polymer. The oven temperature program was as follows: 50°C initially for 1 minute, increased to 145°C at 5°C/min, increased to 175°C at 7°C/min, increased to 195°C at 5°C/min, and then ramped to 250°C at 3°C/min; 250°C was maintained for 10 min. High pure helium (99.999%) was the carrier gas set at a constant flow rate of 1 mL·min^−1^. The injection port, transfer line, and ion source temperatures were all set at 250°C. 70 eV of EI was adopted, and the mass scanning range was set from 50 to 650 amu in full scan. The injection was performed by split mode with a split ratio of 10 : 1. Solvent delay time was set for 3 min for all samples generated by different methods. Xcalibur 2.0 workstation was used to process data.

Most volatile components extracted from ECG were identified by comparing the RIs and comparing the obtained mass spectra of the analytes with those of authentic standards from the NIST libraries (2005) and with the mass spectra published previously [7, 10, 17]. RIs were determined by analyzing a solution containing the homologous series of normal alkanes (C_7_–C_22_) and then calculated as described by van Den Dool and Kratz [18]. Peak areas of all components were calculated by Xcalibur 2.0, and relative amounts (RAs) of volatile compounds were calculated on the basis of peak-area ratios.

## 3. Results and Discussion

### 3.1. Analysis of the Volatile Compounds in ECG

**Figure 1:**
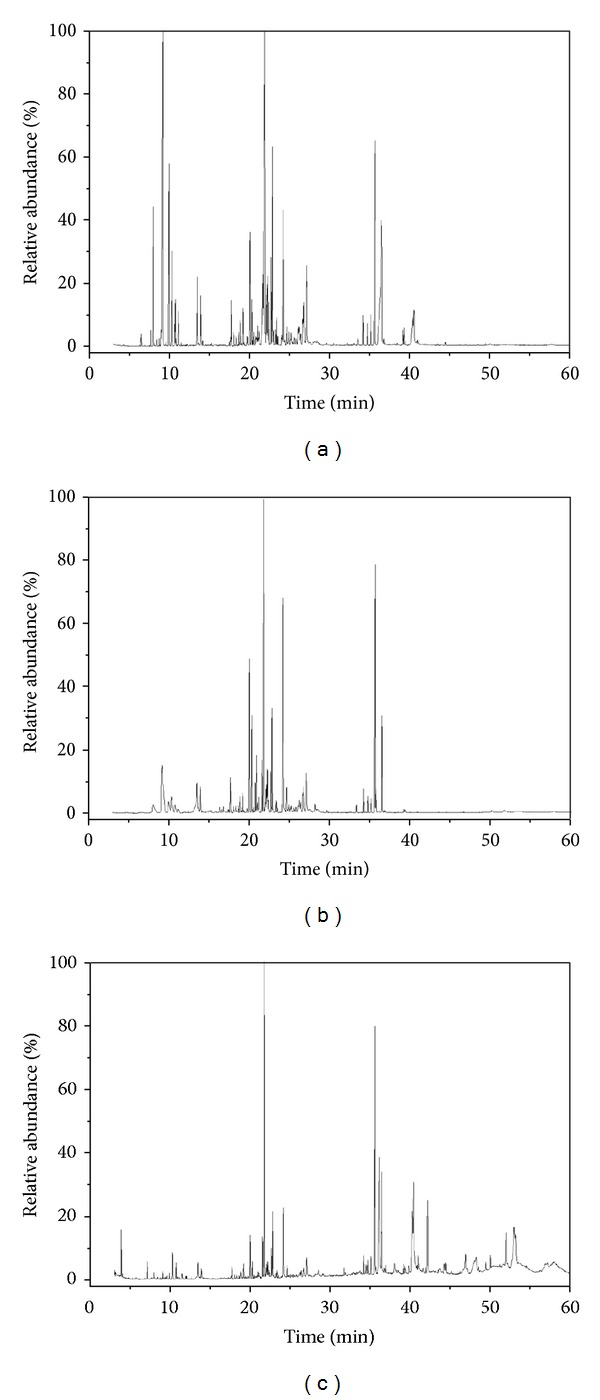
GC/MS total ion chromatograms of ECG by (a) steam distillation, (b) headspace solid-phase microextraction, and (c) solvent extraction.

**Figure 2:**
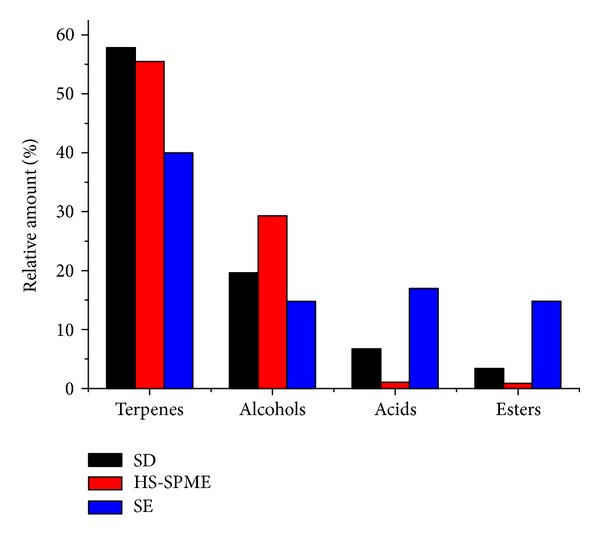
Comparison of volatile categories in ECG by three extraction methods.

The volatile compounds in ECG sample were extracted by HS-SPME, followed by desorption and analysis with GC-MS. The volatile compounds in ECG were also extracted by SD and SE method. The typical total ion chromatograms of the extracts obtained by SD, HS-SPME, and SE were shown in Figure 1 and indicated the differences in volatiles composition among different methods. A total of 81 compounds extracted by SD, HS-SPME, and SE were identified and listed in Table 1, where the RIs and RAs of volatile compounds in ECG were presented. As detailed in Table 1, 77, 56, and 48 compounds were identified by SD-GC/MS, HS-SPME-GC/MS, and SE-GC/MS methods, respectively, and HS-SPME-GC/MS method shared 56 and 34 compounds in common with SD-GC/MS and SE-GC/MS, respectively. The families of detected volatiles in ECG contained terpenes, alcohols, esters, organic acids, ketones, and aldehydes, whose contents by different methods were described in Figure 2. In spite of the three methods, terpenes constituted the most dominant chemical group present in ECG volatiles (39.98~57.81%). It was notable that germacrene-D (the richest terpenoid in ECG volatiles) possessed a larger proportion in ECG, as compared with the previous reports [11, 19]. Second to terpenes, alcohols were another rich common class in ECG volatiles by SD and HS-SPME, whereas acids were the second most constituent in diethyl ether extract. As presented in Table 1 and Figure 2, SD was proved to be efficient in extracting terpenes (57.81%) and alcohols (19.61%), the two families with a relative high content in ECG; SE resulted in a relative high percentage of terpenes (39.98%), acids (16.97%), esters (14.82%), and alcohols (14.76%) with diethyl ether as a solvent. In HS-SPME, the main volatiles using DVB/CAR/PDMS fiber were terpenes (55.47%) and alcohols (29.29%). The results of the three extraction methods indicated that the major volatiles in ECG were terpenes, alcohols, acids, and esters.

SD extracts (yield: 0.89%; yellow-green oil) showed higher proportions of terpenes than those by SPME and SE, not only in the category (38) but also in the relative amount (57.81%). Germacrene-D (13.28%) and limonene (11.77%) were the most two enriched ones followed by 2,6,8,10,14-hexadecapentaene, 2,6,11,15-tetramethyl-, (E,E,E)- (6.54%), *δ-*cadinene (4.73%), *γ*-terpinene (3.75%), *γ-*muurolene (2.87%), *trans*-caryophyllene (2.63%), and *β*-myrcene (2.63%). The major alcohols by SD included *trans-*nerolidol (4.03%), geranyl linalool (3.54%), and *α*-cadinol (2.61%). Acids, mainly hexadecanoic acid (4.79%), could be effectively extracted by SD.

As a moderate and simple extraction method, SE with diethyl ether was also carried out for the extraction of constituents from ECG, yielding 0.11 g of yellow-green viscous concentrates. In the SE extract, terpenes were still the most abundant constituents but much less in category (17) and relative amount (39.98%) than those obtained by the other two methods, which might be due to the evaporation step during the SE process that might lead to the loss of the most volatile components. However, certain high molecular-weight compounds (mainly acids) that did not contribute to the aroma, such as fatty acids, were extracted in large amount (16.97%). Amongst the less volatile components, the relative amount of hexadecanoic acid reached 11.7%, followed by *α*-linoleic acid (4.14%).

The major components of the volatile components extracted by HS-SPME were terpenes (55.47%), which were in accordance with those by SD in categories and percentages. The terpenes by HS-SPME mainly contained germacrene-D (11.36%), 2,6,8,10,14-hexadecapentaene, 2,6,11,15-tetramethyl-, (E,E,E)- (8.49%), limonene (7.49%), *trans*-caryophyllene (5.94%), and so forth. HS-SPME-GC/MS enabled the detection of most odour active compounds in ECG and was indicated to be much richer in alcohols (29.29%) than those obtained by SD (19.61%) and SE (14.76%). The alcohols extracted by HS-SPME were abundant in *trans*-nerolidol (9.9%), terpinen-4-ol (3.42%), and geranyl linalool (3.31%). Nevertheless, compared to SE, HS-SPME was of poor capacity in extracting acids (1.06%) because of the poor volatility and affinity to the fiber of those compounds.

**Figure 3:**
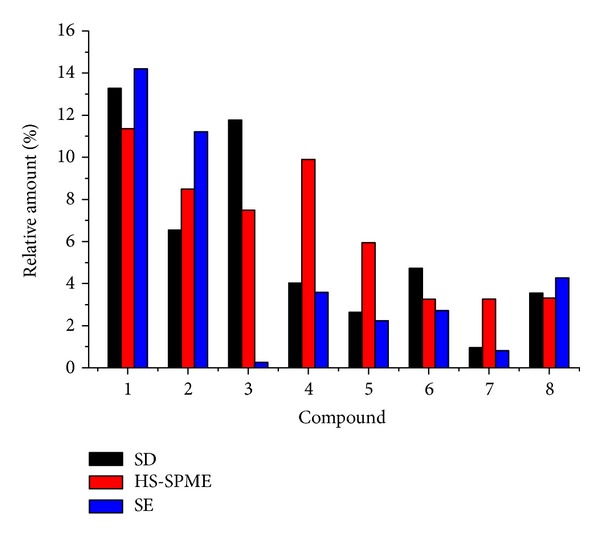
Comparison of the relative amount of the eight target compounds by SD, HS-SPME, and SE methods. (1) Germacrene-D; (2) 2,6,8,10,14-hexadecapentaene, 2,6,11,15-tetramethyl-, (E,E,E)-; (3) limonene; (4) *trans*-nerolidol; (5)* trans*-caryophyllene; (6) *δ*-cadinene; (7) *β*-cubebene; (8) geranyl linalool.

As mentioned above, among all the compounds identified, germacrene-D, 2,6,8,10,14-hexadecapentaene, 2,6,11,15-tetramethyl-, (E,E,E)-, *trans*-nerolidol, and *trans-*caryophyllene accounted for a quite great proportion in total quantity (expressed as RA%).  Figure 3 showed a clear comparison of the RA% values for the eight target compounds extracted by the three methods. The present HS-SPME-GC/MS method obtained much higher RA% for *trans-*nerolidol and* trans-*caryophyllene but lower RA% values for germacrene-D and geranyl linalool. On one hand, this was due to the difference of affinity of the fiber to those compounds. On the other hand, HS-SPME is a relative temperate extraction way especially for those thermally sensitive compounds which could be partly or completely lost during the long-time heating by SD. The comparison among the results by HS-SPME, SD, and SE methods showed that HS-SPME was better for more thermally sensitive volatile compounds, SD for volatile compounds, and SE for high molecular weight compounds. On the whole, the profiles obtained by HS-SPME were similar to those by SD, which revealed that HS-SPME manifested good affinity to principal volatile components of ECG.

### 3.2. Comparison of the Extraction Parameters among the Three Methods

In addition to the amounts of volatile components extracted by the above three methods (i.e., SD, HS-SPME, and SE), other parameters in terms of extraction time, solvent volume, and economic cost about the three extraction methods were also compared. The results were detailed in Table 2. Although it was relatively expensive to purchase the fiber, HS-SPME presented significant advantages over the other two methods in qualitative analysis at analytical level. First, HS-SPME showed significant environmental friendliness compared to SD and SE since it was solvent-free. Then, HS-SPME was clearly fast and efficient (40 min) while 5 h was required for SD and 45 min for SE. Last but not the least, the amount of the plant material used for the HS-SPME analysis was much smaller than that for the SD (36 g) and SE (3 g). The above results indicated that HS-SPME had remarkable advantages in time, plant material, and solvent consuming in rapid extraction and analysis, as compared to the other two methods. However, HS-SPME was inferior to SD and SE in quantitative analysis since it was unattainable for the yield of the crude extract from ECG. 

As one of the most classical extraction techniques for essential oil, SD did not discriminate against most volatiles in the extraction even though it required a long time to accomplish the procedure. Moreover, compared with HS-SPME, SD and SE were more flexible and competent for preparative extraction both in small batches and in large scale.

## 4. Conclusions

In this study, three extraction technologies (SD, HS-SPME, and SE) coupled with GC/MS were compared in terms of the category and the content of volatile components extracted from ECG and other extraction parameters. 77, 56, and 48 volatile components, belonging mainly to terpenes, alcohols, acids, and esters, were extracted and successfully identified, respectively. Germacrene-D and limonene were the two major volatiles in ECG. Amongst the three extraction methods, SD gave a relatively entire profile of volatiles in ECG by long-time extraction; SE enabled the analysis of low volatility and high molecular weight compounds. Both of them were competent for extraction of volatiles at analytical and preparative level. HS-SPME could effectively and rapidly extract principle volatile components from ECG at analytical level, giving similar profiles of volatiles to those by SD. The study indicated that HS-SPME was suitable for rapid qualitative analysis for the volatile components in ECG. This technique could be used for the routine quality control analysis of medicinal plants at analytical level. 

## Figures and Tables

**Table 1:** Volatile compounds of ECG identified by GC/MS.

No.	RI^a^	Name	ID^b^	CAS no.	Relative amount (%)^c^		
					SD	HS-SPME	SE
1	773	1,3,5-Trioxepane	MS, RI	5981-6-6	—	—	1.37
2	834	Furfural	MS, RI	98-01-1	0.05	—	—
3	937	*α*-Pinene	MS, RI	7785-70-8	0.19	—	—
4	960	Heptanol	MS, RI	53535-33-4	—	—	0.94
5	981	*β-*Pinene	MS, RI	18172-67-3	0.27	0.1	—
6	989	*β-*Myrcene	MS, RI	123-35-3	2.63	1.51	0.34
7	1004	*α-*Phellandrene	MS, RI	99-83-2	0.15	—	—
8	1018	*α*-Terpinene	MS, RI	99-86-5	0.14	—	—
9	1022	*o-*Cymene	MS, RI	527-84-4	0.47	—	—
10	1030	Limonene	MS, RI	5989-27-5	11.77	7.49	0.25
11	1045	*trans*-*β*-Ocimene	MS, RI	3779-61-1	0.08	0.05	—
12	1058	*γ-*Terpinene	MS, RI	99-85-4	3.75	1.33	0.23
13	1073	*cis*-Linalool oxide	MS, RI	15249-34-0	1.76	1.84	1.05
14	1082	*α-*Terpinolene	MS, RI	586-62-9	0.35	—	—
15	1086	*trans*-Linalool oxide	MS, RI	34995-77-2	0.67	0.86	0.52
16	1090	*p-*Cymenene	MS, RI	1195-32-0	0.11	—	0.1
17	1102	Linalool	MS, RI	78-70-6	0.52	0.35	—
18	1114	Nonane, 4-ethyl-5-methyl-	MS, RI	1632-71-9	—	—	0.19
19	1140	Cosmene	MS, RI	460-01-5	0.04	—	0.3
20	1175	Terpinen-4-ol	MS, RI	20126-76-5	1.2	3.42	0.69
21	1190	*α-*Terpineol	MS, RI	98-55-5	0.91	1.47	0.4
22	1206	Decanal	MS, RI	112-31-2	0.1	0.05	—
23	1239	Carvone	MS, RI	99-49-0	0.05	0.11	—
24	1290	Thymol	MS, RI	89-83-8	0.04	0.32	—
25	1299	Carvacrol	MS, RI	499-75-2	0.05	0.37	—
26	1306	Undecanal	MS, RI	112-44-7	0.04	0.07	—
27	1335	*ο*-Elemene	MS, RI	3242-08-8	0.15	1.06	—
28	1339	*δ-*Elemene	MS, RI	20307-84-0	0.69	0.73	0.28
29	1351	*α-*Cubebene	MS, RI	17699-14-8	0.23	0.31	0.14
30	1362	*cis-*Geranyl acetate	MS, RI	141-12-8	0.21	0.41	0.1
31	1376	Ylangene	MS, RI	14912-44-8	0.21	0.25	0.12
32	1378	*α-*Copaene	MS, RI	3856-25-5	0.7	0.66	0.45
33	1395	*β*-Elemene	MS, RI	515-13-9	0.72	0.74	0.59
34	1411	Dodecanal	MS, RI	112-54-9	0.25	0.2	0.09
35	1417	*trans*-Caryophyllene	MS, RI	87-44-5	2.63	5.94	2.23
36	1420	*β-*Cubebene	MS, RI	13744-15-5	0.95	3.26	0.81
37	1433	*β-*Aromadendrene	MS, RI	25246-27-9	0.23	0.15	—
38	1451	Isoledene	MS, RI	NA^d^	0.23	1.06	—
39	1455	1,4,7,-Cycloundecatriene, 1,5,9,9-tetramethyl-, Z,Z,Z-	MS, RI	NA^d^	0.36	0.28	—
40	1463	2-Isopropenyl-4a,8-dimethyl-1,2,3,4, 4a,5,6,7-octahydronaphthalene	MS, RI	NA^d^	0.28	0.75	—
41	1480	*γ-*Muurolene	MS, RI	30021-74-0	2.87	2.11	1.68
42	1486	Germacrene-D	MS, RI	23986-74-5	13.28	11.36	14.2
43	1494	*α-*Muurolene	MS, RI	10208-80-7	1.07	1.92	1.12
44	1511	*γ-*Cadinene	MS, RI	39029-41-9	1.62	1.35	1.2
45	1528	*δ-*Cadinene	MS, RI	483-76-1	4.73	3.25	2.71
46	1536	Naphthalene, 1,2,3,4,4*α*,7-hexahydro-1,6-dimethyl-4-(1-methylethyl)-	MS, RI	16728-99-7	0.29	0.25	0.17
47	1541	*α-*Cadinene	MS, RI	24406-05-1	0.49	0.39	0.27
48	1552	Calacorene	MS, RI	38599-17-6	0.2	0.16	—
49	1561	*trans-*Nerolidol	MS, RI	40716-66-3	4.03	9.9	3.58
50	1572	(−)-Spathulenol	MS, RI	77171-55-2	0.47	1.14	0.63
51	1583	Globulol	MS, RI	51371-47-2	0.42	0.38	—
52	1600	Viridiflorol	MS, RI	552-02-3	0.55	0.37	—
53	1620	*β-*Eudesmol	MS, RI	473-15-4	0.24	0.29	—
54	1641	Cubenol	MS, RI	21284-22-0	0.67	1.29	—
55	1647	Selina-6-en-4-ol	MS, RI	1461-03-6	0.32	0.52	—
56	1654	*τ-*Candinol	MS, RI	5937-11-1	0.56	—	—
57	1661	*τ-*Muurolol	MS, RI	19912-62-0	1.47	2.14	1
58	1668	*α*-Cadinol	MS, RI	481-34-5	2.61	2.29	1.06
59	1683	Ledene oxide-(II)	MS	NA^d^	0.17	—	0.24
60	1730	5,6,6-Trimethyl-5-(3-oxobut-1-enyl)-1-oxaspiro[2.5]octan-4-one	MS	NA^d^	0.08	0.15	—
61	1840	Cyclopentadecanone, 2-hydroxy-	MS, RI	4727-18-8	0.08	—	—
62	1857	Pentadecanoic acid	MS, RI	1002-84-2	0.22	0.3	—
63	1923	1,3,6,10-Cyclotetradecatetraene	MS, RI	1898-13-1	0.53	0.75	0.85
64	1927	Kaur-16-ene, (8*β*,13*β*)-	MS	20070-61-5	0.41	0.61	0.62
65	1933	Hexadecanoic acid, methyl ester	MS, RI	112-39-0	0.63	0.53	0.9
66	1946	2,6,8,10,14-Hexadecapentaene, 2,6,11,15-tetramethyl-, (E,E,E)-	MS	38259-79-9	6.54	8.49	11.21
67	1975	Hexadecanoic acid	MS, RI	57.10.3	4.79	0.07	11.7
68	2008	Geranyl linalool	MS, RI	1113-21-9	3.54	3.31	4.27
69	2023	Bergapten	MS, RI	484-20-8	0.13	—	1.33
70	2079	Methyl linoleate	MS, RI	112-63-0	0.31	0.13	0.47
71	2102	Methyl linolenate	MS, RI	301-00-8	0.44	0.2	—
72	2139	Osthole	MS, RI	484-12-8	0.03	—	0.53
73	2150	Z,Z-10,12-Hexadecadien-1-ol acetate	MS, RI	60-33-3	1.53	—	3.12
74	2158	*α-*Linolenic acid	MS, RI	463-40-1	1.56	—	4.14
75	2177	Ethyl linoleate	MS, RI	7619-08-1	0.11	—	—
76	—	*α-*Glyceryl linolenate	MS	18465-99-1	0.16	—	0.84
77	—	Isogeijerin	MS	38409-25-5	—	—	5.21
78	—	3-Ethyl-5-(2-ethylbutyl)-octadecane	MS	55282-12-7	0.06	—	—
79	—	Heptadecane, 9-hexyl-	MS	55124-79-3	0.07	—	—
80	—	2,2′-Methylenebis(6-tert-butyl-*p*-cresol)	MS	119-47-1	0.04	—	1.13
81	—	Auraptene	MS	495-02-3	0.06	—	2.41

^a^Retention indices were calculated using a homologous series of *n*-alkanes (C_7_–C_22_).

^b^Identification of volatile compounds was carried out by comparing MS spectrum and RIs of components in ECG with those of the authentic standards in NIST library (2005) and previous study. In the comparison of MS spectrum, the requisites should be that both SI and RSI were more than 800.

^c^Results obtained by peak-area normalization.

^d^NA: not available.

**Table 2:** Comparison of extraction parameters among the three extraction methods for volatiles from ECG.

	SD	HS-SPME	SE
Extraction time (min)	300	40	45
Solvent	Water	—	Diethyl ether
Solvent volume (mL)	300	None	90 (30∗3)
Material amount (g)	36	0.2	3
Yield (%)	0.89	NA^a^	3.67
Cost ^b^	++	+++	+

^a^Not available.

^b^The degrees of the economic cost were expressed by “+”.
